# Development of a Fatal Noncompressible Truncal Hemorrhage Model with Combined Hepatic and Portal Venous Injury in Normothermic Normovolemic Swine

**DOI:** 10.1371/journal.pone.0108293

**Published:** 2014-09-24

**Authors:** Ujwal R. Yanala, Jason M. Johanning, Iraklis I. Pipinos, Gustavo Larsen, William H. Velander, Mark A. Carlson

**Affiliations:** 1 Department of Surgery, University of Nebraska Medical Center, Omaha, Nebraska, United States of America; 2 Department of Vascular Surgery, University of Nebraska Medical Center, Omaha, Nebraska, United States of America; 3 Department of Genetics, Cell Biology and Anatomy, University of Nebraska Medical Center, Omaha, Nebraska, United States of America; 4 Department of Surgery, VA Nebraska–Western Iowa Health Care System, Omaha, Nebraska, United States of America; 5 Department of Chemical and Biomolecular Engineering, University of Nebraska–Lincoln, Lincoln, Nebraska, United States of America; Georgia Regents University, United States of America

## Abstract

Noncompressible truncal hemorrhage and brain injury currently account for most early mortality of warfighters on the battlefield. There is no effective treatment for noncompressible truncal hemorrhage, other than rapid evacuation to a surgical facility. The availability of an effective field treatment for noncompressible truncal hemorrhage could increase the number of warfighters salvaged from this frequently-lethal scenario. Our intent was to develop a porcine model of noncompressible truncal hemorrhage with a ∼50% one-hour mortality so that we could develop new treatments for this difficult problem. Normovolemic normothermic domestic swine (barrows, 3 months old, 34–36 kg) underwent one of three injury types through a midline incision: 1) central stellate injury (N = 6); 2) excision of a portal vein branch distal to the main PV trunk (N = 6); or 3) hemi-transection of the left lateral lobe of the liver at its base (N = 10). The one-hour mortality of these injuries was 0, 82, and 40%, respectively; the final mean arterial pressure was 65, 24, and 30 mm Hg, respectively; and the final hemoglobin was 8.3, 2.3, and 3.6 g/dL, respectively. Hemi-transection of the left lateral lobe of the liver appeared to target our desired mortality rate better than the other injury mechanisms.

## Introduction

The two major causes of early mortality during modern military operations have been (1) hemorrhage and (2) traumatic brain injury [1], [2], [3], [4]. It has been estimated that >80% of battlefield deaths in the modern era are secondary to uncontrolled hemorrhage [3], [5], [6], [7], [8]; moreover, recognition of vascular injury during the U.S. conflicts in Iraq and Afghanistan occurred with five-fold greater frequency than in earlier wars [9]. About half of these hemorrhagic battlefield deaths involved noncompressible torso injury or bleeding from an area not conducive to tourniquet control [2], [6], [10], [11]. In battlefield fatalities classified as potentially survivable, noncompressible truncal hemorrhage was the cause of death in ∼50% [3], [8].

Most cases of compressible hemorrhage (e.g., bleeding from extremity trauma) can be controlled in the field with currently-available methods (e.g., direct compression and/or tourniquet), allowing for safe transport to a forward surgical care unit [12]. No effective field therapy currently exists, however, for hemorrhage from a noncompressible truncal injury [8], [13], [14]. So in order to have a chance at survival, a warfighter with a bleeding truncal injury requires immediate evacuation to a forward surgical care unit for emergency operative intervention. In modern warfare with extremely mobile combat elements, however, the time required for transport of an injured combatant to a forward surgical unit may extend to several hours [15]. Given the rapid clinical course of uncontrolled truncal hemorrhage, development of a field therapy which could be administered by a first-responder within minutes of wounding might improve survival from torso injury [3], [8], [14].

Most studies that have focused on the development of advanced hemostatic devices have utilized porcine models of compressible injury at various sites (e.g., aorta, femoral vessels, and liver) [13], [16], [17]. A few reports describing noncompressible intraabdominal hemorrhage models intended for development of local treatment have been published, including rat [18], rabbit [19] and, most recently, one model in swine [13], [20], [21], [22]. In order to study site-directed (i.e., intraabdominal) therapies for noncompressible truncal hemorrhage, we have developed our own model of noncompressible truncal hemorrhage involving combined hepatovenous and portovenous injury in resuscitated domestic swine. In the present report we describe and characterize this porcine model of noncompressible truncal hemorrhage, compare it to other models, and discuss how it may be utilized to develop novel field therapies for noncompressible intraabdominal bleeding.

## Materials and Methods

### Animal Welfare

This animal research study was carried out in accordance with recommendations in the *Guide for the Care and Use of Laboratory Animals* (8^th^ ed.) from the National Research Council and the National Institutes of Health [23], and also in accordance with the Animal Welfare Act of the United States (U.S. Code 7, Sections 2131 – 2159) [24]. The animal protocol was approved by the Institutional Animal Care and Use Committee of the VA Nebraska-Western Iowa Health Care System (protocol number 00760), by the Institutional Animal Care and Use Committee of the University of Nebraska Medical Center (protocol number 11-064-07-ET), and by the Animal Care and Use Review Office of the United States Army Medical Research and Materiel Command (award number W81XWH-11-1-0836). All procedures were performed in animal facilities approved by the Association for Assessment and Accreditation of Laboratory Animal Care International (AAALAC; www.aaalac.org) and by the Office of Laboratory Animal Welfare of the Public Health Service (http://grants.nih.gov/grants/olaw/olaw.htm). All surgical procedures were performed under isoflurane anesthesia, and all efforts were made to minimize suffering. Euthanasia was performed in accordance with the AVMA Guidelines for the Euthanasia of Animals [25]. In addition, this study was conceived, designed, documented, analyzed, and reported per the ARRIVE (Animal Research: Reporting of In Vivo Experiments) Guidelines [26]; the ARRIVE Guidelines Checklist is shown in Table S1 in file S2.

### Determination of Subject Numbers, Study Design, and Randomization

The minimum number of swine (n = 6) utilized in each group in the development of a noncompressible hemorrhage model was determined with a statistical power analysis [27] using Δ/σ (Cohen's *d*, in which Δ is the desired difference in means set by the observer, and σ is the estimated standard deviation) = 2.0, false positive rate (α) = 0.05, false negative rate (β) = 0.2, and power (1−β) = 0.8. Due to the heuristic nature of the studies in this report, the animal subjects were not randomized. The intent of this study was to explore the effect of various injury mechanisms in normothermic normovolemic swine in order to obtain a reasonable model of noncompressible truncal hemorrhage. A specific injury was tested in pre-determined minimum number of subjects (six; see Methods); if that injury did not generate a reasonable outcome (i.e., a ∼50% one-hour mortality), then experimentation proceeded to another injury type. Although it may have been theoretically possible to randomize subjects among the injury mechanisms tested, such a randomization would have assumed at the outset that at least one mechanism was going to have the desired mortality rate; otherwise the study goal would not have been met. Since we could not rely on this assumption during the initial planning of this study, we decided to proceed with an iterative explorative study design in order to discover an appropriate injury mechanism.

### Animal Preparation

Domestic swine (castrated males, age 3 months) were purchased from the Agricultural Research and Development Center (Mead, NE) of the University of Nebraska–Lincoln. Subjects underwent an acclimatization period of at least four days, during which time they underwent veterinary examination and daily observation to confirm good health. Subjects were fed ad lib with corn-soybean meal supplemented with vitamins, and maintained in SPF (specific-pathogen free) conditions. Each subject was fasted for 12 hours before the surgical procedure, but with free access to water.

Animal preparation followed a previous description [28]. Each subject was premedicated with a single 3 mL IM injection containing 150 mg Telazol (tiletamine hydrochloride and zolazepam hydrochloride, 1∶1 by weight; Fort Dodge Animal Health, New York, NY), 90 mg ketamine, and 90 mg xylazine (drugs were combined in saline immediately prior to injection). After premedication each subject was weighed, and then intravenous line was established in an auricular vein. Oral endotracheal intubation (7.5 mm internal diameter cuffed tube) was performed, and anesthesia was maintained with 0.5–1.5% isoflurane using a Matrx Model 3000 Veterinary Anesthesia Ventilator (Midmark Corp., Versailles, OH). Mechanical ventilation was maintained at 12–15 breaths per minute, with a tidal volume of 10–15 mL/kg, in order to keep the end-tidal pCO_2_ at 30–40 mm Hg. A heating pad was placed under each subject to support body temperature. A cutdown in the right neck (along the medial edge of the sternocleidomastoid muscle) was performed, and then a carotid arterial catheter (20 gauge) was inserted for pressure monitoring and blood sampling, followed by a jugular venous catheter (16 gauge) for isotonic fluid administration. Arterial pressure, end-tidal pCO_2_, rectal temperature, cardiac electrical activity, and pulse oximetry (tongue probe) were continuously recorded with a Bionet BM5 Veterinary Monitor (Bionet America, Inc.; Tustin, CA) interfaced to a laptop computer. Each swine subject was maintained under an appropriate level of isoflurane anesthesia (indicated by absence of the corneal reflex) for the duration of the procedure; prior to euthanasia, the isoflurane was increased (see below).

Upon placement of the arterial line, 20 mL of blood was withdrawn for a serum test set, which included a complete blood count (CBC), protime (PT), partial thromboplastin time (PTT), international normalized ratio (INR), fibrinogen, arterial blood gas analysis (ABG), and thromboelastography (TEG). After completion of the above preparations, a ventral midline laparotomy incision was made through the linea alba, starting at the xiphoid process and extending inferiorly. Just superior to the urethral meatus, this incision was angled to the right in order to avoid the midline penis and urethra (but medial to the nipple line), and then was continued inferiorly down to but not into the right groin. The incision was performed with cautery to control any bleeding points from the musculoaponeurotic layers. Splenectomy then was performed, followed by placement of a cystostomy tube (18 French Foley) in the dome of the urinary bladder, secured with a purse string silk suture. The cystostomy tube exited through a stab incision in the left lower quadrant, and was connected to gravity drainage. The excised spleen was weighed, and then a volume of warm Lactated Ringers (LR; 37°C) solution equivalent to three-fold the splenic weight was administered through the jugular line, using a rapid infusion pump (Cole-Palmer Masterflex L/S; Vernon Hills, IL) set at 150 mL/min. An improvised intraabdominal pressure (IAP) monitor (100 mL IV bag) then was placed along the left paracolic gutter; the pressure line of this monitor exited out of the superior end of the laparotomy incision, and was connected to the Bionet monitor (kept level with the subject) for continuous recording of IAP. The IAP monitor was zeroed while the abdominal incision was open.

Prior to injury, any blood loss incurred during the preparation was quantified by weighing tared surgical sponges that were used to absorb lost blood, and then a volume of LR equivalent to three-fold the pre-injury blood loss (typically <50 mL) was given using the infusion pump. Immediate pre-injury vital signs were recorded, the lower half of the midline incision was closed with towel clips, and then one of three primary injury mechanisms was applied as described below.

### Injury Mechanisms

All injuries were performed in normothermic normovolemic (resuscitated) swine; only one injury was performed per subject. Immediately after injury, the abdominal incision was closed rapidly with towel clips in all subjects (Figure S1 in file S1). No post-injury treatment (other than fluid resuscitation; see below) was administered.

#### 1. Central Liver Injury (CLI)

The porcine normothermic normovolemic stellate liver laceration model was adapted from previous descriptions [28], [29]. A liver laceration was created with a custom-built liver injury clamp [28], which consisted of metal tines in an X-configuration (5 cm diameter) on one arm of the clamp, and a base plate on the other arm onto which the tines seated. The base plate was placed on the inferior surface of the liver against the quadrate lobe, between the cystic duct and the portal vein. The tines were positioned over the liver dome, directly anterior (within 1 cm) to the vena cava at the base of the left medial hepatic segment. The clamp then was closed, forcing the tines through the liver dome and onto the base plate. The clamp immediately was re-opened, moved 2–3 cm to the right, and then closed again, such that the second clamp strike overlapped ∼50% with the first strike.

#### 2. Portal Vein Resection (PVR)

**Figure 1:**
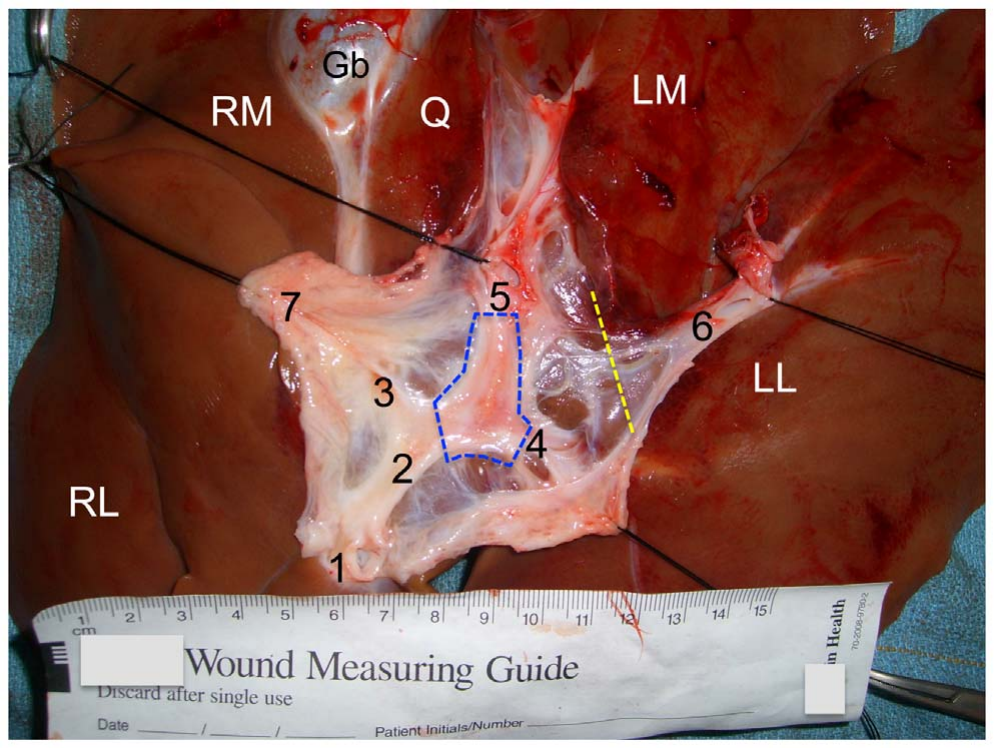
Dissection demonstrating the anatomy of the porcine intrahepatic portal venous system. *Ex vivo* porcine liver, inferior aspect (scale in cm). The soft tissues overlying the portal venous system have been dissected and retracted with silk stay sutures. RL = right lateral lobe; RM = right medial lobe; LM = left medial lobe; LL = left lateral lobe; Q = quadrate lobe; Gb = gallbladder; 1 = cut orifice of main portal vein; 2 = intrahepatic portal vein; 3 = RM lobe portal vein branch; 4 = 1^st^ LL lobe portal vein branch; 5 = cut orifice of 2^nd^ LL lobe portal vein branch (proximal end); 6 = distal end of structure 5; 7 = pedicle containing the common bile duct and hepatic artery (reflected laterally by stitch). In this dissection the 2^nd^ LL lobe portal vein branch was transected (the two ends are labeled as 5 and 6). The hepatic veins were not exposed in this dissection. The dashed blue polygon indicates the portion of the portal vein that was resected for the PVR injury mechanism. The dashed yellow line indicates where the cut was made across base of LL lobe for the LLLH injury mechanism. Scale = cm. [201 words].

In this injury, the left main branch of the portal vein was resected. The inferior surface of the liver was exposed by lifting the right medial and left medial lobes superiorly. The position of the left main branch of the portal vein was superficial and readily visible, running in the fissure between the left medial and left lateral lobes of the liver. Using sharp dissection and a right angle clamp, the left main branch of the portal vein was encircled and then controlled with a silk ligature. The silk ligature was placed on anterior traction, and a ∼2 cm segment of the left main branch then was excised with 2–3 rapid cuts of a short curved Mayo scissors. The portal vein branch was transected just proximal to the silk ligature, the cutting then continued in the plane beneath the vein, and the excision was completed where the left main branch of the portal vein split into the left medial and left lateral lobes of the liver (Figure 1 and Figure S2 in file S1).

#### 3. Hepatic Left Lower Lobe Hemitransection (LLLH)

**Figure 2:**
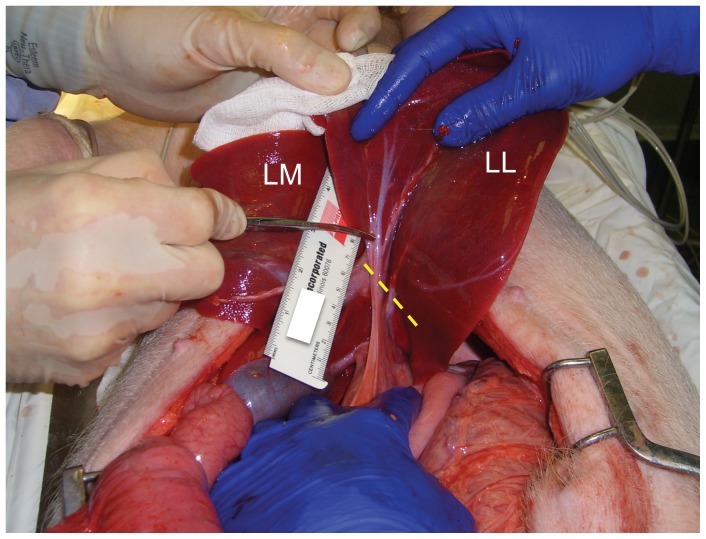
Operative set-up for the porcine noncompressible hemorrhage model. View of the open abdomen in a living anesthetized pig, looking toward the head. The left medial (LM) and left lateral (LL) lobes of the liver have been exteriorized through a ventral midline incision. The dashed yellow line indicates the location of the imminent cut across base of LL lobe. The tips of the scissors are touching the 2^nd^ LL lobe portal vein branch (refer to Figure 1).

Hemitransection of the left lateral lobe of the liver was performed by first elevating this lobe anteriorly into the operative field (Figure 2). The base of the left lateral lobe was identified with gentle finger pinching, and then confirmed visually. A short curved Mayo scissors then was placed across the base of the left lower lobe, directed posteriorly, but taking care not to incorporate any of the left medial lobe. A single cut across the base of the left lower lobe of the liver was performed using the full length of the scissors' blade (∼4 cm); see Figures 1 and 2. The liver lobe then was allowed to drop back into the abdominal cavity.

### Post-injury Management

As mentioned above, the remainder (upper half) of the laparotomy incision was closed immediately after the liver injury with towel clips, which required <30 s. This produced a completely closed abdomen after injury creation. No attempt at hemorrhage control (compression, bandage application, vessel clamping, etc.) was performed for any of the injuries in this report. All injuries were intended to be negative (i.e., no-treatment) controls in order to document the natural course of hemorrhage from a noncompressible truncal injury.

The planned post-injury observation period was 60 min. When the mean arterial pressure (MAP) dropped below the resuscitation target pressure (defined as 80% of the pre-injury MAP), resuscitation was initiated with rapid infusion (150 mL/min) of warm LR (one liter bags stored in a 37°C incubator) into the jugular venous line. This resuscitation was maintained as long as the MAP was below the target, but was limited to 100 mL/kg (e.g., 3.5 L for a 35 kg pig). Isoflurane was maintained at 0.5–1%, as long as the subject did not demonstrate any signs of wakefulness (such as twitching or >1 spontaneous breaths between ventilator cycles).

### Euthanasia

If the subject survived the 60 min post-injury observation period, then the isoflurane was increased to 5%, the incision was opened, and all procedural blood loss was evacuated from the abdomen. After 5% isoflurane had been administered for 5 min, a transverse incision was made in the diaphragm, widely opening both pleural spaces, and the supradiaphragmatic inferior vena cava was transected. Such exsanguination under anesthesia is an AVMA-approved method of euthanasia [25]. If the subject did not survive the 60 min observation period, then the above diaphragmatic incision and caval transection were performed after all procedural blood loss was evacuated from the abdomen.

### Endpoints

Heart rate, MAP, pulse oximetry, end-tidal pCO_2_, rectal temperature were continuously recorded (see Figure S3 in file S1), as described above. Continuous recording of intraabdominal pressure was incorporated into the experimental protocol toward the end of the study, on subjects injured with LLLH. A second serum test set was drawn 15 min after injury; a third test set was drawn at 60 min or just prior to expiration, whichever occurred first. Death was defined as MAP ≤10 mm Hg, no identifiable pressure wave on the monitor's arterial tracing, and end-tidal pCO_2_<5 mm Hg. Immediately after the 60 min observation period or after the animal expired (whichever came first), the laparotomy incision was re-opened, and all clots and blood were rapidly evacuated into tared buckets with a combination of tared laparotomy pads, suction, and manual extraction. The buckets were weighed in order to calculate blood loss. Gross necropsy then was performed, including removal of the liver for inspection, dissection, photography, and documentation of injury anatomy. In the selection process of an appropriate injury for a noncompressible truncal hemorrhage model, a target of 50% one-hour mortality was chosen empirically, according the to rationale outlined in the Introduction.

### Laboratory Testing

The CBC, PT/PTT, INR, fibrinogen, and ABG testing were contracted to the Clinical Laboratory of the VA Nebraska-Western Iowa Health Care System. This laboratory used the quantitative fibrinogen assay based on the von Clauss method [30]. Thromboelastography was performed with a TEG 5000 Thrombelastograph (Haemonetics Corp.; Braintree, MA) as previously described [28], [31],with some modifications. Whole blood (340 µl) was incubated at 37°C and mixed with 200 mM CaCl_2_ (20 µl). The thromboelastograph was calibrated each day of use. Each time point of each analysis was run in triplicate. TEG Analytical Software (version 4.2.2) was used to calculate the time to clot initiation (R), time to clot firmness of 20 mm (K), alpha angle (α), maximal clot strength (MA, which was directly related to the shear elastic modulus strength, G), and percent lysis 60 minutes after MA (LY60) [32].

### Statistical Analysis

Numerical data is reported as the mean ± standard deviation (SD). Groups of numerical data were compared with the nonparametric Kruskal-Wallis analysis of variance. Groups of categorical data were compared with the Fisher exact test. Significance was defined as p<0.05.

## Results

A total of 23 swine underwent injury in this study in the following order: CLI, N = 6; PVR, N = 6; splenic pedicle transection, N = 1; and LLLH, N = 10. The first injury mechanism to be evaluated for the noncompressible hemorrhage model was CLI, which involved a stellate laceration through the dome of the liver immediately anterior to the inferior vena cava (Figure S4 in file S1). This injury mechanism has been utilized in porcine hemorrhage models since the 1990's [29]. The severity of the hemorrhage from this injury appears to be dependent on laceration of at least two major hepatic veins as they enter the intrahepatic vena cava [29]; refer to Figure S5 in file S1. In order to ensure that at least two major hepatic veins were cut, two overlapping strikes of the injury clamp were applied directly anterior to where the inferior vena cava enters the liver (see Methods). Incidentally, the towel clip incisional closure utilized in the experiments of this report could withstand a sustained intraabdominal insufflation pressure of 70 mm Hg without leakage of gas or fluid (Figure S1 in file S1).

**Figure 3:**
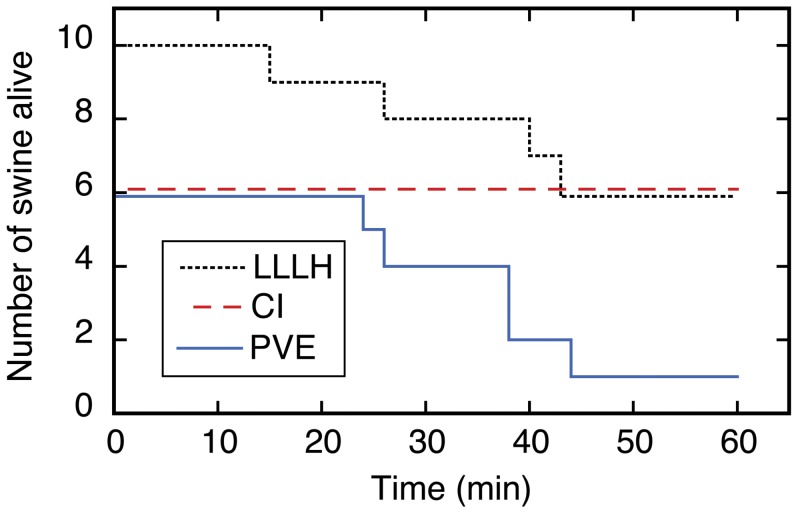
Kaplan Meier survival plot for the three models of noncompressible hemorrhage. Time zero = moment of injury; CLI = central liver injury (N = 6); PVR = portal vein resection (N = 6); LLLH = hepatic left lower lobe hemitransection (N = 10).

**Figure 4:**
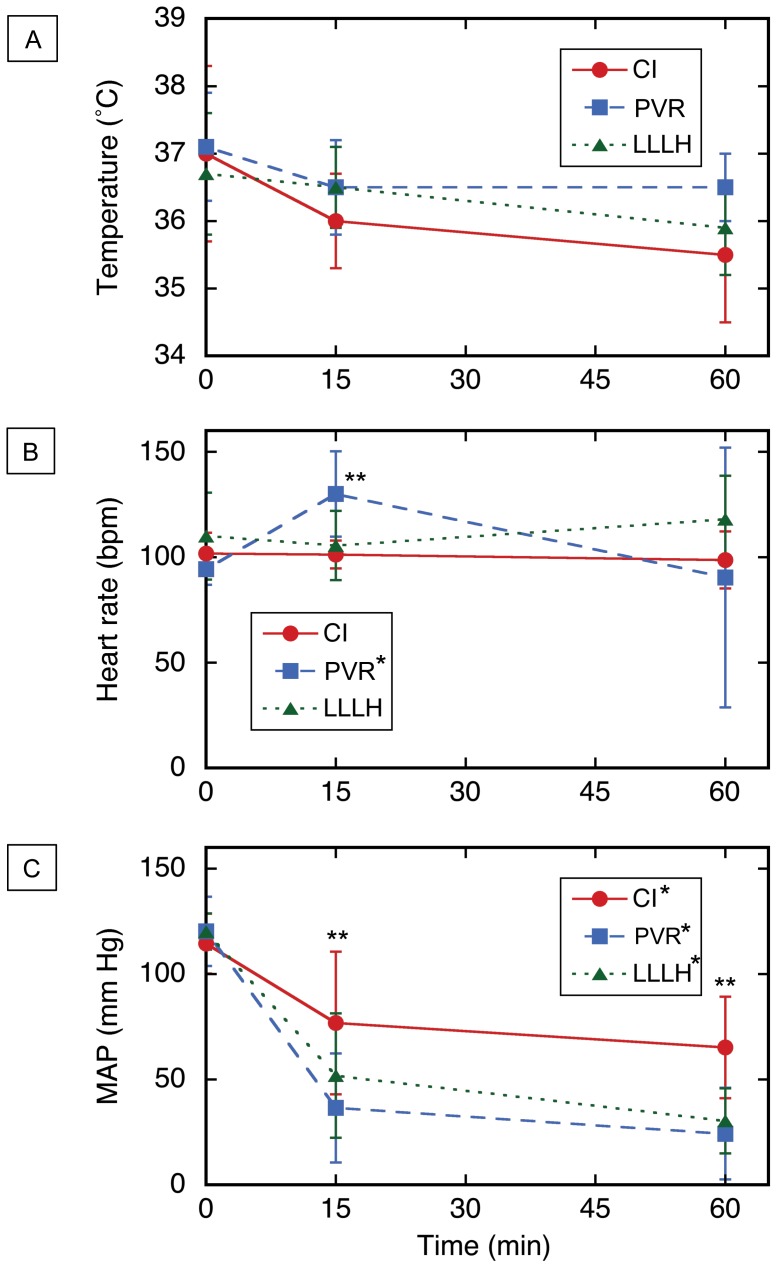
Vital sign data for three porcine models of noncompressible truncal hemorrhage. (A) Temperature. (B) Heart rate. (C) Mean arterial pressure (MAP). Time zero = moment of injury; CLI = central liver injury (N = 6); PVR = portal vein resection (N = 6); LLLH = hepatic left lower lobe hemitransection (N = 10). Values shown are mean ± sd; *p<0.05, Kruskal–Wallis one-way analysis of variance on all three time points for a given injury; **p<0.05, Kruskal–Wallis one-way analysis of variance on all three injuries at the indicated time point. Also refer to Table S2 in file S2.

**Figure 5:**
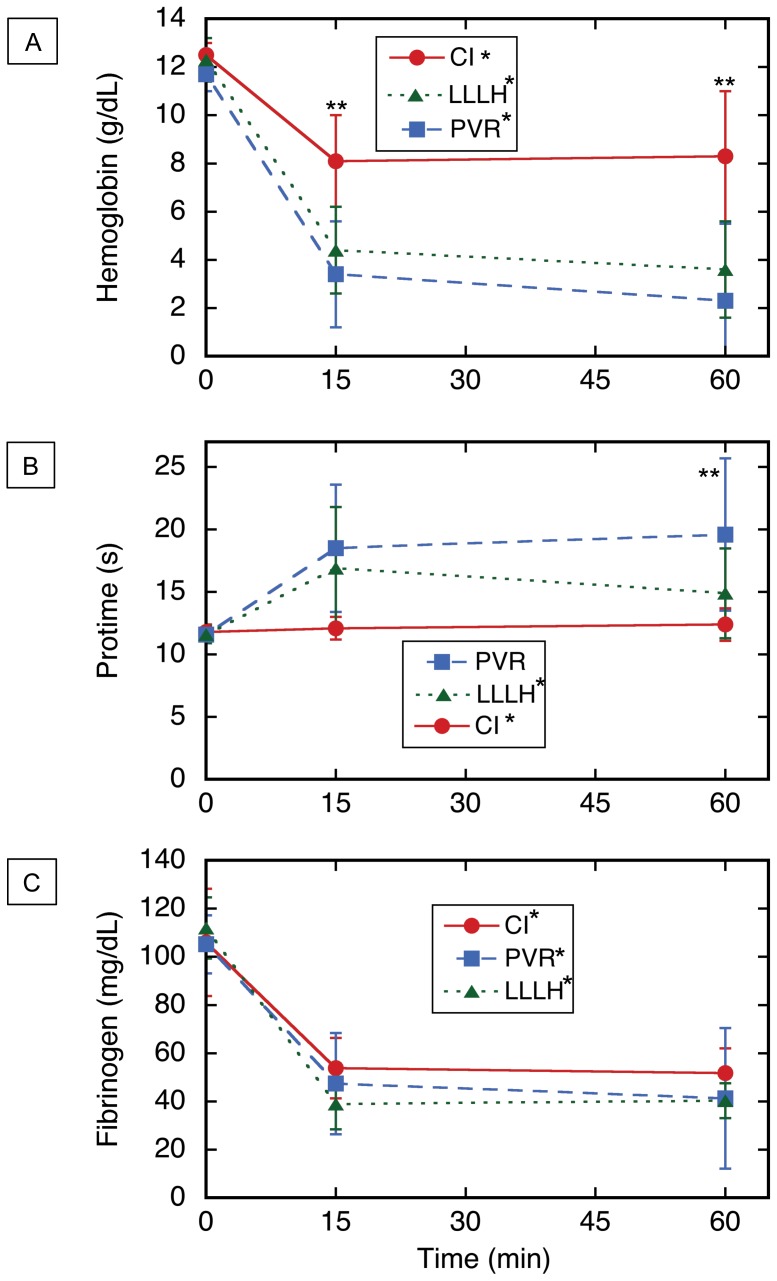
Hematologic testing for three porcine models of noncompressible truncal hemorrhage. (A) Serum hemoglobin. (B) Protime. (C) Serum fibrinogen. Time zero = moment of injury; CLI = central liver injury (N = 6); PVR = portal vein resection (N = 6); LLLH = hepatic left lower lobe hemitransection (N = 10). Values shown are mean ± sd; *p<0.05, Kruskal–Wallis one-way analysis of variance on all three time points for a given injury; **p<0.05, Kruskal–Wallis one-way analysis of variance on all three injuries at the indicated time point. Also refer to Table S2 in file S2.

CLI was performed on six consecutive subjects as described above, and the 60 min mortality was zero (Figure 3 and Table 1). Most of the subjects survived easily, with an average MAP of 65 mm Hg at 1 h after injury (Figure 4 and Table 1). Mean blood loss after CLI was ∼1 L; the hemoglobin dropped ∼4 g/dL, the platelet count decreased ∼100 K, the fibrinogen level decreased by ∼50%, and the base excess dropped from ∼2 to about zero mmol/L. There was no significant effect on other parameters or laboratory tests (Figure 5, Figure S6 in file S1, and Tables S2, S3, and S4 in file S2). At necropsy, it was found that the injury site had sealed against the diaphragm in all subjects, which produced hemostasis of the untreated CLI (Figure S4 in file S1). Dissection of the explanted liver (Figure S4 in file S1) confirmed that 1–4 hepatic veins (median 3.0) were lacerated (defined as a cut involving more than half of the vessel circumference) in each subject (Table S4 in file S2). Since the one-hour mortality for the untreated CLI injury was zero after six subjects, it was concluded that the CLI mechanism in swine would not be sufficiently lethal for a noncompressible hemorrhage model.

**Table 1:** Select endpoints (at time of death or 1 hr post-injury) for the three injury mechanisms of noncompressible hemorrhage.

Injury Type	Survival at 1 h, N (%)	Median survival time (min)	MAP, mm Hg	Blood loss, mL	Hemoglobin, g/dL	Base excess, mmol/L	Protime, s
1. CLI	6/6 (100)	68	65±24	1140±684	8.3±2.7	0.7±1.5	12.4±1.3
2. PVR	1/6 (18)	38	24±22	3142±1121	2.3±3.2	−4.9±10.5	19.6±6.1
3. LLLH	6/10 (60)	65	30±16	2853±657	3.6±2.0	−3.6±7.3	14.9±3.6
p-value	**0.0025** *	**0.0382** †	**0.0192** †	**0.0042** †	**0.0084** †	0.4146†	**0.0298** †

CLI = central liver injury; PVR = portal vein resection; LLLH = hepatic left lateral lobe hemitransection; MAP = mean arterial pressure. Continuous data expressed as mean ± standard deviation.

*Categorical data compared with the Fisher Exact Test;

† continuous data compared with the Kruskal-Wallis nonparametric analysis of variance.

The second injury mechanism tested was resection of the left main branch of the portal vein segment (Figures 1 and S2 in file S1). Six swine underwent such a resection, and only one was alive 1 h after injury (Figure 3). The other five subjects died from exsanguination 24–44 min after injury, with an average blood loss of 3.1 L, a nine point drop in hemoglobin, an increase in protime to ∼20 s, a ∼60% drop in fibrinogen, a decrease in bicarbonate from 29 to 16 mmol/L, and final MAP <30 mm Hg (Figures 4 and 5, Figure S6 in file S1, Table 1, and Table S4 in file S2). There were no significant changes in thromboelastographic parameters (Table S3 in file S2). At necropsy, all subjects had ongoing hemorrhage from the injury site. *Ex vivo* dissection of the liver (Figure S2 in file S1) confirmed excision of the left main branch of the portal vein in all six subjects. Since nearly all subjects died prior to the one-hour endpoint, it was concluded that resection of the left main branch of the portal vein was too lethal for a noncompressible truncal hemorrhage model.

Incidentally, splenic pedicle transection was performed in a single subject. This was done by transecting the splenic pedicle 1 cm proximal to the mass silk ligature (securing both the splenic artery and vein) placed during the splenectomy. This subject survived the 1 h observation period easily, with only 651 mL of blood loss. At necropsy, this injury had clotted, and there was no active hemorrhage. Based on the experience with the CLI mechanism, it was decided not to pursue the splenic pedicle transection any further. This single subject is mentioned here for completeness.

**Figure 6:**
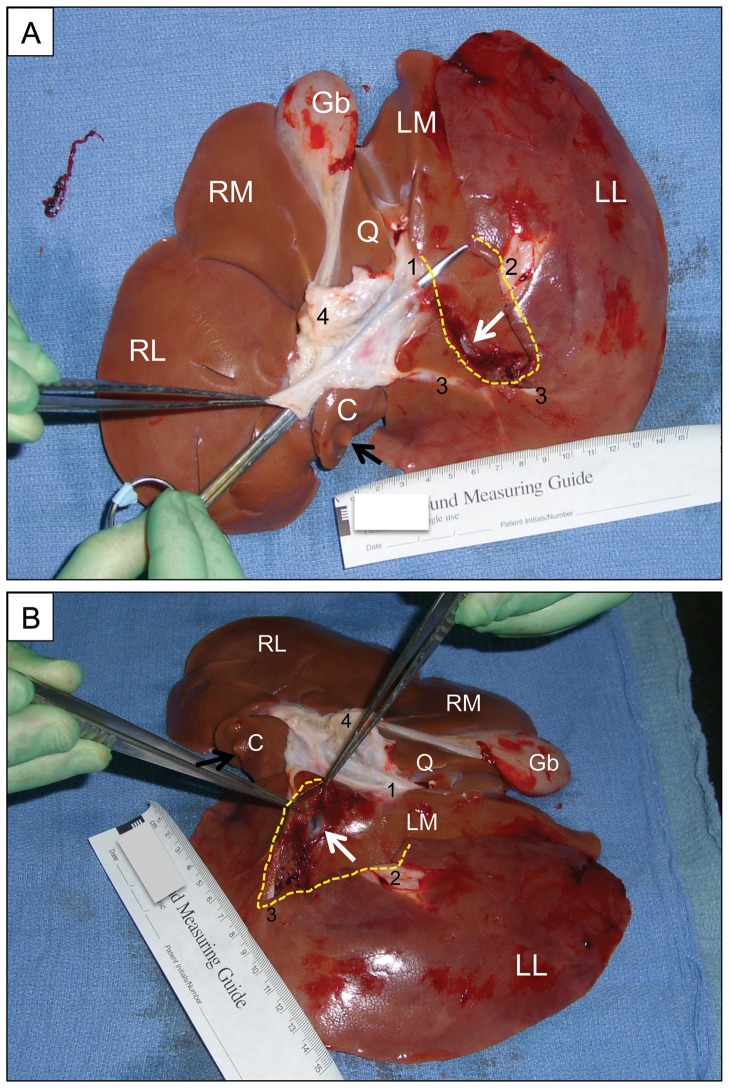
Postmortem liver ex vivo, demonstrating the standard injury. (A) Inferior aspect; anterior toward top of image. (B) Left inferior oblique aspect; anterior toward right of image. Scale in cm. RL = right lateral lobe; RM = right medial lobe; LM = left medial lobe; LL = left lateral lobe; Q = quadrate lobe; C = caudate lobe (black arrow indicates location of infrahepatic IVC); Gb = gallbladder. The scissors in panel A has been inserted through the cut orifice of the main portal vein, and the scissors tip are emerging through the transected 2^nd^ portal vein branch to the LL lobe (1); the distal end of this portal vein branch also is indicated (2). The gap in the liver parenchyma created by the injury is indicated with a dashed yellow line. The 1^st^ portal vein branch to the LL lobe (3), visible at the bottom of the wound, was not injured in this subject. The soft tissues (including the common bile duct and hepatic artery) overlying the portal venous system have been dissected and flipped anteriorly (4). White arrow indicates orifice of transected hepatic vein to the LL lobe; the latter has a dusky appearance relative to the other lobes.

The third and final injury mechanism tested for the noncompressible hemorrhage model was hemitransection of the left lateral lobe of the liver near its base, or LLLH (Figures 1, 2, and 6; also see the Video in File S3 for a demonstration of this injury mechanism). Ten subjects were injured with this mechanism, and four expired prior to the one hour endpoint (Figure 3). Continuous vital sign tracings for a typical surviving and nonsurviving subject are shown in Figure S3 in file S1. The final average MAP was 30 mm Hg (Figure 4 and Table 1); the MAP of the six surviving subjects was 39 mm Hg (Table S2 in file S2). The average blood loss for all ten subjects was 2.8 L, and the derangements in hemoglobin, BE, and INR were intermediate between the central liver injury group (minimal effect) and the portal vein resection group (excessive effect); see Figure 5, Figure S6 in file S1, Table 1, and Table S4 in file S2. There appeared to be changes in the thromoboelastographs of the LLLH subjects from pre- to post-injury (Figure S7 in file S1), but the derived parameters (R, K, α, and MA) were not significantly different (Table S3 in file S2). Dissection of the explanted liver (Figure 6) demonstrated that the median number of portal vein branch and hepatic vein transections were 1.0 (range 1–2) and 1.0 (range 0–1), respectively, with seven of ten subjects demonstrating transection of one portal vein branch (the 2^nd^ branch supplying the left lateral lobe) and one large (>7 mm) hepatic vein (draining the left lateral lobe).

The pre-injury heart rate, mean arterial pressure, rectal temperature, hemoglobin, protime, PTT, INR, fibrinogen, thromboelastography, pH, pO_2_, pCO_2_, and bicarbonate were not statistically different among the three injury groups (Figures 4 and 5, Figure S6 in file S1, and Tables S2 and S3 in file S2). Pre-injury platelet count, pre-injury base excess, pre-injury blood loss, and pre-injury fluid administration, were not equivalent among the three injury groups (Table 2). The mean splenic mass increased ∼100 g over the course of this study (Table 2), while subject weight did display significant variation (Table S4 in file S2).

**Table 2:** Pre-injury parameters not equivalent among the three injury groups.

Injury Type	Pre-injury platelet count (1,000/µL)	Pre-injury base excess (mmol/L)	Pre-injury blood loss (mL)	Pre-injury fluid (mL)	Splenic Wt (g)
1. CLI	368±52	2.3±1.3	289±48	917±122	231±47
2. PVR	409±68	4.9±1.7	339±71	930±156	261±50
3. LLLH	288±61	3.5±2.6	374±37	1160±176	335±34
*p-value	**0.0134**	**0.0379**	**0.0238**	**0.0043**	**0.0014**

CLI = central liver injury; PVR = portal vein resection; LLLH = hepatic left lateral lobe hemitransection. Data expressed as mean ± standard deviation;

*Kruskal-Wallis nonparametric analysis of variance.

Pre-injury blood loss includes the full mass of the spleen plus any incidental blood loss incurred during subject preparation for injury. Pre-injury fluid = intravenous isotonic crystalloid administered prior to injury.

## Discussion

As discussed above, the reasonable outcome that was being sought in this study was a ∼50% mortality within the first hour after injury. This mortality rate goal was set empirically, based upon the observations of severe noncompressible truncal hemorrhage in a military setting. Furthermore, we believed that an injury mechanism with either a higher or lower one-hour mortality probably would not allow us to discriminate differences in efficacy in subsequent comparisons of experimental treatments for noncompressible truncal hemorrhage. That is, if the injured subject bled too “slow” or too “fast,” then future comparison of treatment regimens might be meaningless.

We believe that domestic swine are a good choice to model severe hemorrhage in humans because, by the age of 3 months, domestic swine have reasonably large size (∼35 kg) and blood volume (∼2.6 L [33]) which makes studies of severe hemorrhage practical. We have access to an inbred population of domestic swine that has been closed for >30 years, which theoretically should reduce inter-subject variability. Furthermore, domestic swine have been used for decades to model human physiology and pathophysiology (including hemorrhage and hemostasis), and generally have produced acceptable data for these types of studies [16], [17]. Although small animal models (e.g., rabbits and rodents) have produced some usable data in the field of hemostasis [18], [19], information obtained from small animal models of hemorrhage ultimately may have limited clinical relevance because of their small organ size, blood vessel diameter, and blood volume with respect to humans.

A rabbit model of noncompressible hemorrhage was described in which partial hepatectomy (liver edge resection) was combined with a systemic administration of a Factor X inhibitor [19]. A fibrin sealant foam therapy was demonstrated to have hemostatic efficacy in this model compared to both placebo treatment or no-treatment controls. A model of noncompressible hemorrhage involving portovenous injury in domestic swine also has been described [13], [20], [21]. In this model, the investigators looped wires around the medial liver lobes through a midline laparotomy, and then transected these lobes by pulling the wires out of the abdomen after the incision had been closed. The subjects then were resuscitated with crystalloid with no limit on resuscitation volume. The one-hour mortality of this injury model was 90%, with a median survival time of 43 min. This group of investigators subsequently described a therapy for noncompressible hemorrhage consisting of an expansile polyurethane foam, which had demonstrable efficacy in their porcine model [20], [21]. The same investigator group described another porcine model of noncompressible hemorrhage which utilized placement of a wire around the external iliac artery via a laparotomy, and subsequent transection of the artery by wire distraction after the abdominal incision had been closed [22]. One-hour mortality in this model was 78%, with a median survival time of 32 min.

Use of a “closed” abdominal technique (i.e., incision not left open) makes empiric sense in the design of a noncompressible injury model, since clinical intraabdominal hemorrhage from blunt or penetrating trauma occurs within a system that essentially is closed. Therefore, the model should be able to mimic conditions that develop in such a closed system, such as increased intraabdominal pressure, massive release/activation of clotting factors, and poor accessibility for compression-based interventions. The noncompressible model described in the present report accomplishes this goal in that a closed system is created immediately after injury by rapid closure (towel clipping) of the midline incision. The previous reports of a porcine noncompressible hemorrhage models [13], [20], [21], [22] also accomplished this goal, as the laparotomy incision in that model was closed at the time of injury.

In comparison to the recently-published swine models of noncompressible torso hemorrhage, our model had a more moderate one-hour mortality (40%), but produced similar decreases in blood pressure and hemoglobin. The hepatovenous/portovenous injury in our model was conceptually similar to that of the wire-distraction model [13], though the technique of inducing the injury obviously was different between these two models. We also utilized routine pre-injury splenectomy (see below discussion), not done in the above studies. Our subject size was 5–10 kg smaller than used in the above studies. We limited our resuscitation volume to 100 mL/kg of crystalloid (or 3.5 L in a 35 kg subject, given at 150 mL/min, with a resuscitation target of 80% of the pre-injury MAP). The resuscitation fluid limit in the other described porcine portovenous noncompressible injury model was 10 L (given at 100 mL/min) with a resuscitation target MAP of 65 mm Hg [13], [20], [21]; in this group's description of a porcine noncompressible iliac artery injury model, however, the crystalloid resuscitation was limited to 1 L (two 500 mL boluses within the first 20 min) [22].

Of note, previous animal [34] and clinical studies [35] have suggested that in subjects with uncontrolled hemorrhage and no immediate operative intervention, hypotensive resuscitation (variably defined, but typically meaning administration of very little or no intravenous fluid) will increase survival. The concept of hypotensive resuscitation has been discussed in the literature since the early 1900's [36]. Although studies on the clinical benefits of hypotensive resuscitation have not been uniformly positive [37], the current Tactical Combat Casualty Care (TCCC) guidelines recommend the use of hypotensive resuscitation (<1 L of 6% hetastarch) in prehospital management of uncontrolled hemorrhage, and then early use of 1∶1 blood products (1 unit of plasma transfused with every unit of packed red blood cells) in conjunction with hemorrhage control in the surgical unit [5], [38], [39], [40], [41]. The question of whether “low” vs. “high” volume crystalloid resuscitation will produce better survival in our porcine noncompressible model was not addressed in the present study. Regarding 1∶1 blood product utilization, we and the other investigators [13], [20], [21], [22] developing these porcine models of “battlefield” noncompressible, uncontrolled hemorrhage have not employed this type of resuscitation; some investigators, however, have infused salvaged autologous whole blood in similar models [42].

Routine splenectomy during the pre-injury preparation of porcine hemorrhage models has been a common but controversial practice [43]. It has been argued that the contractile porcine spleen can participate in an “auto-transfusion” phenomenon during severe blood loss [44], which may have a confounding effect on the subject's response to massive blood loss. While routine splenectomy is a commonly-practiced preparatory technique in porcine models of severe hemorrhage, the utilization of splenectomy is not universal in this type of research [43], [45]. Obviously, a trauma victim does not undergo splenectomy with fluid replacement prior to incurring a torso injury; the practice of pre-injury splenectomy in swine hemorrhage models may introduce other effects that are not entirely understood [43]. Of note, it was not our intent with the present study to determine whether splenectomy should or should not be performed as a component of these hemorrhage models.

The meaning of the difference in the pre-injury platelet counts was not clear, as pre-injury platelet count means were all within normal limits. Similarly, the meaning of the difference in the pre-injury base excess values probably was not relevant, in that these values also were within normal limits, and none of the subjects demonstrated signs of hypovolemia or under-resuscitation prior to injury. The meaning of the difference in splenic mass among the three injury groups was not clear; this simply may have been a random event. The differences in pre-injury blood loss (which included spleen mass) and pre-injury fluid administration could be explained by the difference in splenic mass among the three injury groups (Table 2), because the splenic mass directly influenced both the pre-injury blood loss and pre-injury fluid administration (see Methods).

The primary utility of a large animal model of noncompressible intraabdominal hemorrhage is, of course, to develop treatments for this difficult clinical problem. An expansile polyurethane foam has been demonstrated to have efficacy in the wire-induced portovenous injury model of noncompressible hemorrhage [21]. This foam is nonresorbable, and was associated with brief periods of marked intraabdominal hypertension (>100 mm Hg) after injection, followed by a plateau of intraabdominal pressure at ∼30 mm Hg [21]. While effective, there has been some concern for foam-induced pressure injury to the intestines in these studies [20], [21]. Using the model of the present report, we currently are developing an alginate-based foam supplemented with an optimized human fibrin sealant [28] for treatment of noncompressible hemorrhage (see Figure S8 in file S1). This foam formulation is fully-resorbable, and has not been associated with excessive intraabdominal pressure in preliminary experiments (unpublished data).

The observation of inter-group differences in some preoperative descriptors (Table 2) was unexpected. Although it could be argued that some of these differences were not relevant, the inter-group difference in splenic weight is hard to explain. It is possible that the domestic swine, which were drawn from a large, closed (inbred) population of inbred domestic swine were not as uniform as initially assumed. Alternatively, the difference in splenic weight among the three injury groups simply could be ascribed as a chance result (i.e., a false positive), which theoretically should occur once in every 20 statistical tests, if the α is set at 0.05. The actual effect of the differing splenic weights on the noncompressible hemorrhage in this study was not clear.

## Conclusions

A porcine model of noncompressible torso hemorrhage was developed which involved a combined hepatic vein and portal vein transection. The model had a one hour mortality of 40%. This model should be useful for development of field therapies for uncontrolled hemorrhage after truncal hemorrhage, which currently accounts for a large proportion of battlefield deaths among warfighters. An effective therapy for uncontrolled truncal hemorrhage may reduce battlefield mortality.

## Supporting Information

File S1 **Figures S1–S8.**
(PDF)

File S2 **Tables S1–S4.**
(ZIP)

File S3 **Video.**
(MP4)
