# Supplementary material for: Development of a Fatal Noncompressible Truncal Hemorrhage Model with Combined Hepatic and Portal Venous Injury in Normothermic Normovolemic Swine
Source: PLoS One. 2014 Sep 24;9(9):e108293. doi: 10.1371/journal.pone.0108293 (PMC4176969; doi:10.1371/journal.pone.0108293)
Supplement: File S1 — Figures S1–S8. (PDF) [file pone.0108293.s001.pdf]

S1-S8 Figure Legends

Figure S1. Incisional closure with towel clips. (A) Overhead view of the porcine abdomen shortly after creation of a hepatic left lower lobe hemitransection. The subject is supine, with the head to the right of the image. The ventral midline laparotomy incision is shown closed with towel clips. The spacing between clips is between 5-10 mm. 1 = transabdominal cystostomy tube. (B) Lateral viewe of a different porcine subject ~20 min after creation of a hepatic left lower lobe hemitransection. The ventral midline laparotomy incision was closed with towel clips as in panel A, and then the abdomen was insufflated with oxygen to ~70 mm Hg. No leakage of gas or blood occurred through the incision. The old blood present on along the flank and abdomen was smeared there during the preparatory phase. Note the expansion of regions outside of the peritoneal cavity (e.g., the groin, indicated with arrows), presumably secondary to gaseous dissection of soft tissue planes (i.e, emphysema). 1 = oxygen insufflation line (traverses the midline incision); 2 = transabdominal cystostomy tube. Subject is supine; cephalad is to the right of the image.

Figure S2. Portal vein resection in a normothermic normovolemic swine. *Ex vivo* liver (inferior aspect) from a subject that expired from excision of a portal vein segment, showing the anatomy of the injury. The injury was created by excising the portal vein branches to the left medial and left lateral lobes. The tip of the forceps indicates the proximal transected end of the left portal vein supplying the left side of the liver; the dashed yellow curves indicate the approximate positions of the excised branches of the left portal vein (one to the left medial lobe and two to the left lateral lobe). The hepatic artery branches also were presumed to have been excised, though they were not identified in these dissections. Markers: C = caudate lobe of liver; CBD = open end of common bile duct; Gb =

gallbladder; IVC = inferior vena cava; LL = left lateral lobe of liver; LM = left medial lobe of liver; PV = open end of portal vein; RL = right lateral lobe of liver; RM = right medial lobe of liver. Scale = cm.

Figure S3. Continuous vital sign data from two typical tests of noncompressible hemorrhage. (A) Subject expired from exsanguination prior to 60 min endpoint. (B) Subject survived beyond 60 min endpoint, then euthanized. Injury mechanism in both subjects was hepatic left lower lobe hemitransection mechanism, done at t = 0. Splenectomy with fluid replacement done at t minus ~15 min.

Figure S4. Central liver injury in a normothermic normovolemic swine. The injury was created with two applications of 5 cm scissoring tines (liver injury clamp; see Methods) through the hepatic dome, immediately anterior to the IVC, as described in the methods. (A) Upon re-opening the midline incision 1 h after injury, the dome of the liver (containing the injury site) was adherent to the diaphragm with clot (small black arrows). There was very little free blood in the abdomen. (B) Similar subject at 60 min; the liver has been pulled off the diaphragm, revealing a large clot (small black arrows) covering the injury site. (C) Superior aspect of liver *ex vivo* from subject in panel A. Injury site is encircled with dashed yellow line. Remnant of hemostatic clot is shown by small black arrows, forming a rim around the injury. (D) Superior aspect of liver *ex vivo* from another subject with similar injury, showing interior of injury (dashed yellow oval). The forceps have been inserted through the open end of the suprahepatic IVC; the forceps tips protrude out of the lacerated hepatic vein to the left medial lobe (retracted by the lower hand). Markers: measure bar = 5 cm; large black arrow = cephalad; D = diaphragm; Gb = gallbladder; H = surgeon hand; LL = left lateral lobe of liver; LM = left medial lobe of liver; RL = right lateral lobe of liver; RM = right medial lobe of liver; S = stomach; Y = Yankauer suction.

Figure S5. Hepatic venous drainage in the swine. Posterior-superior aspect of the *ex vivo* porcine liver. The organ was explanted by transecting the inferior vena cava just above and below the liver, cutting the diaphragmatic and retroperitoneal attachments, and then cutting across the porta hepatis. The intrahepatic IVC then was opened along its longitudinal axis on the posterior aspect, exposing the interior of the intrahepatic IVC. The edges of the incised IVC have been retracted with silk stay sutures in order to splay the vessel open. This exposure affords view of the anterior aspect of the inside of the IVC, so that the venous drainage from the liver into the IVC can be demonstrated. The visible portion of the liver in this image is primarily the dome. The dashed yellow line indicates the suprahepatic end of the opened IVC, where it was transected to explant the organ. The orifices of the hepatic veins to the left lateral (LL), left medial (LM), right medial (RM), and right lateral (RL) liver lobes are indicated with numerals 1-4, respectively. Small tributaries from the liver entering the IVC directly are indicated with small white arrows. Medial scale = cm.

Figure S6. Arterial blood gas values for three porcine models of noncompressible truncal hemorrhage. (A) Arterial pH. (B) Partial pressure of carbon dioxide (pCO_2). (C) Serum bicarbonate (HCO_3^-). (D) Base excess. Time zero = moment of injury; CLI = central liver injury (N = 6); PVR = portal vein resection (N = 6); LLLH = hepatic left lower lobe hemitransection (N = 10). Values shown are mean ± sd; *$p < 0.05$, Kruskal–Wallis one-way analysis of variance on all three time points for a given injury; **$p < 0.05$, Kruskal–Wallis one-way analysis of variance on all three injuries at the indicated time point. Also refer to Table S2.

Figure S7. Sample TEG tracing in a subject with noncompressible hemorrhage. Injury mechanism was hepatic left lower lobe hemitransection mechanism; subject survived the 60 min observation period. Refer to Table S3 for statistical analysis of TEG data.

Figure S8. Demonstration of intraabdominal foam injection. (A) Overhead view of subject's ventral aspect. Alginate-based foam (~3 L) was injected into the subject's abdomen (no liver injury), the midline incision was closed with towel clips, and the incision was re-opened for this image 1 h later. Cephalad is to the right. Ruler is in in/cm. (B) Extraction of foam from subject in panel A, taken several min after the image in panel A. View is from the subject's head, looking caudad. Firmness and tensile strength of foam allowed its removal as one intact mass. Arrow = line of IAP monitor caught in the foam mass.

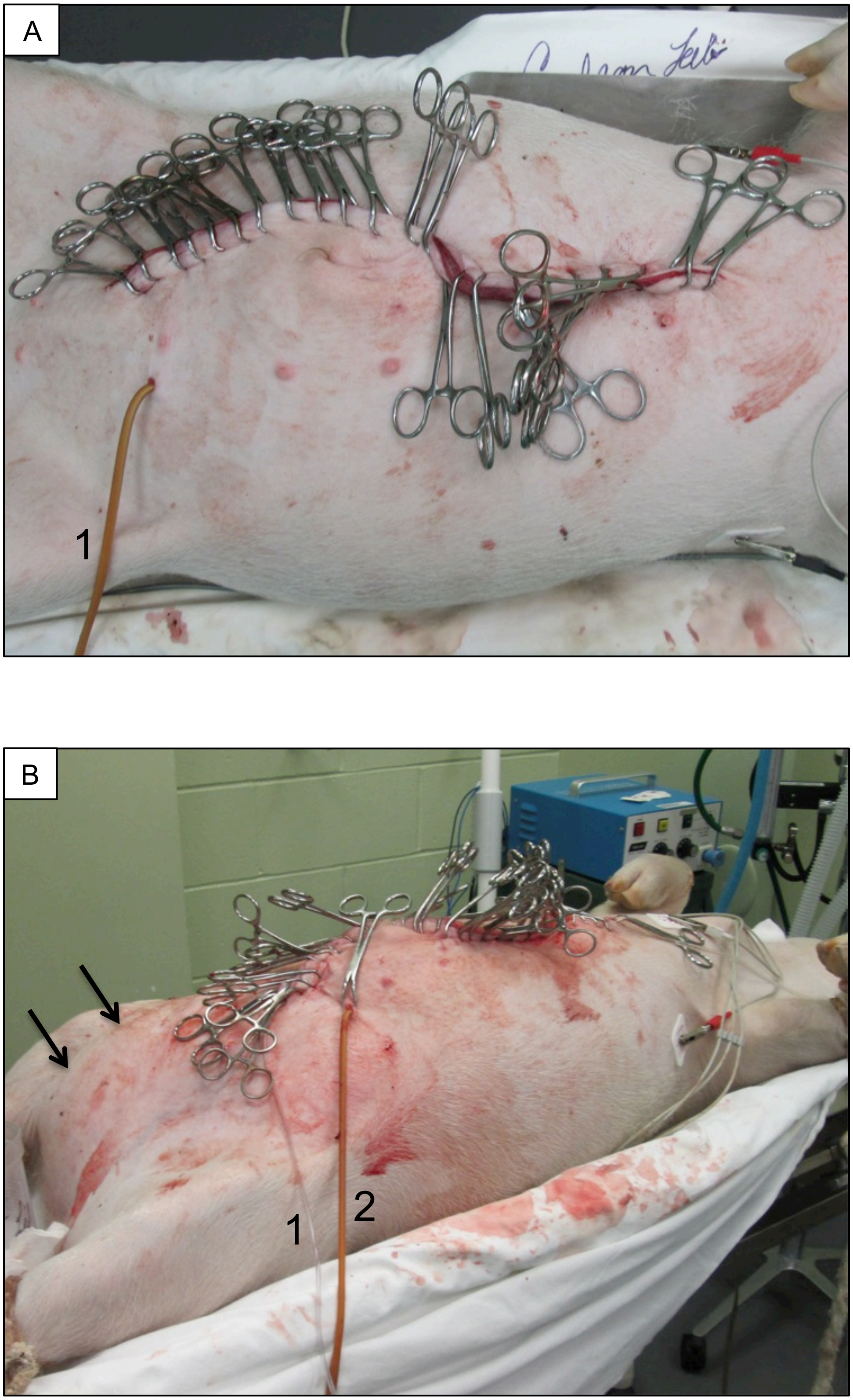

Figure S1, Yanala et al.

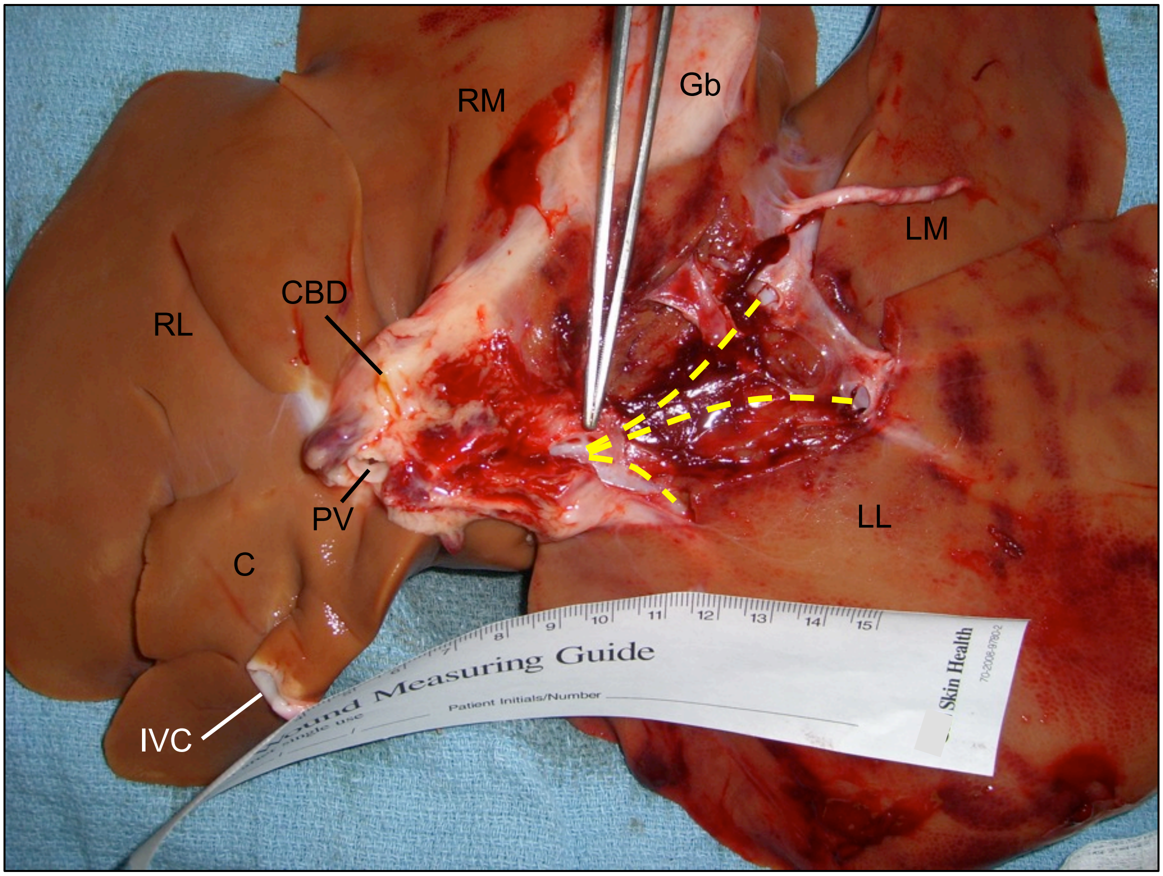

Figure S2, Yanala et al.

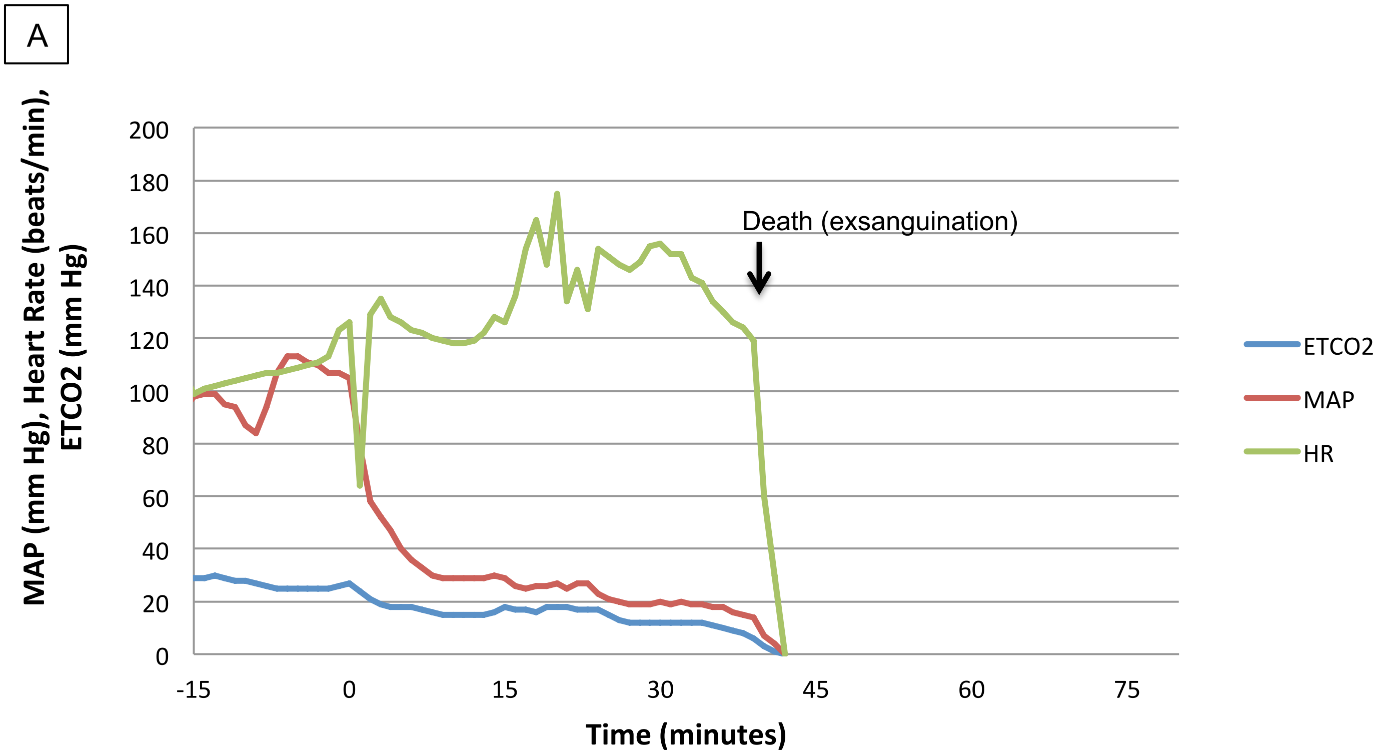

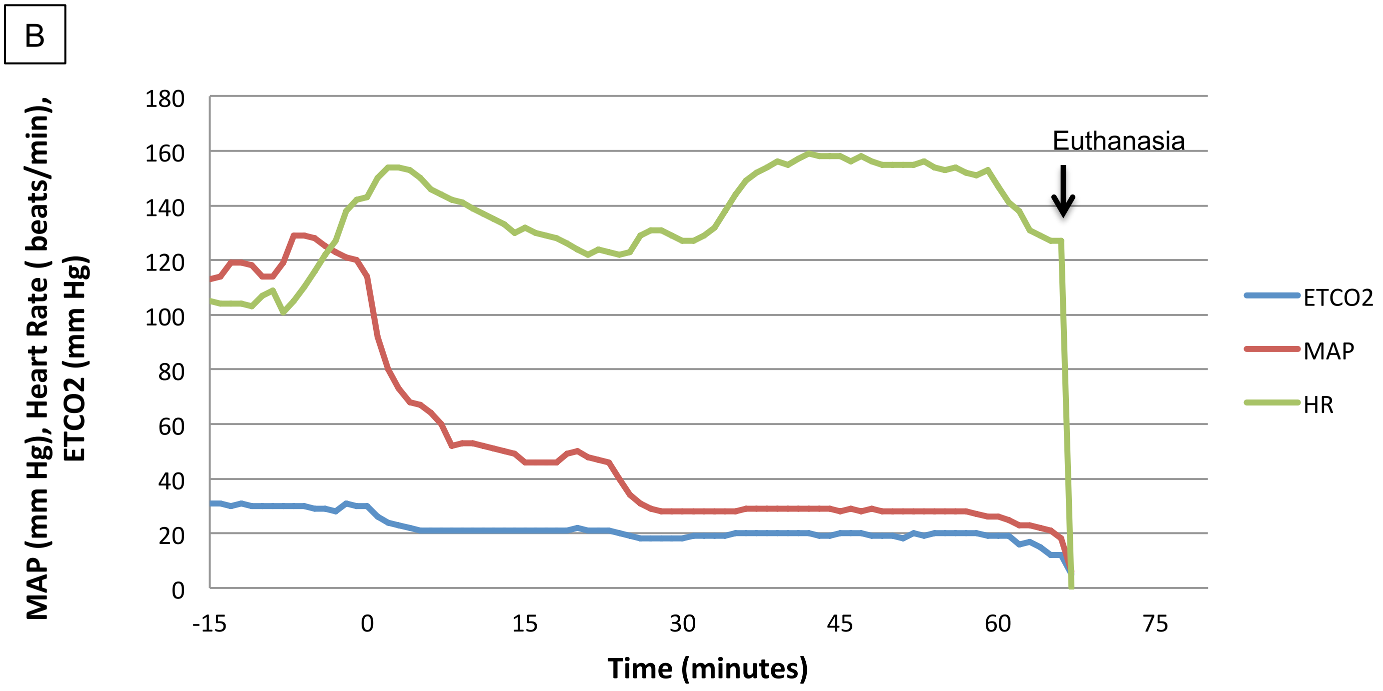

Figure S3, Yanala et al.

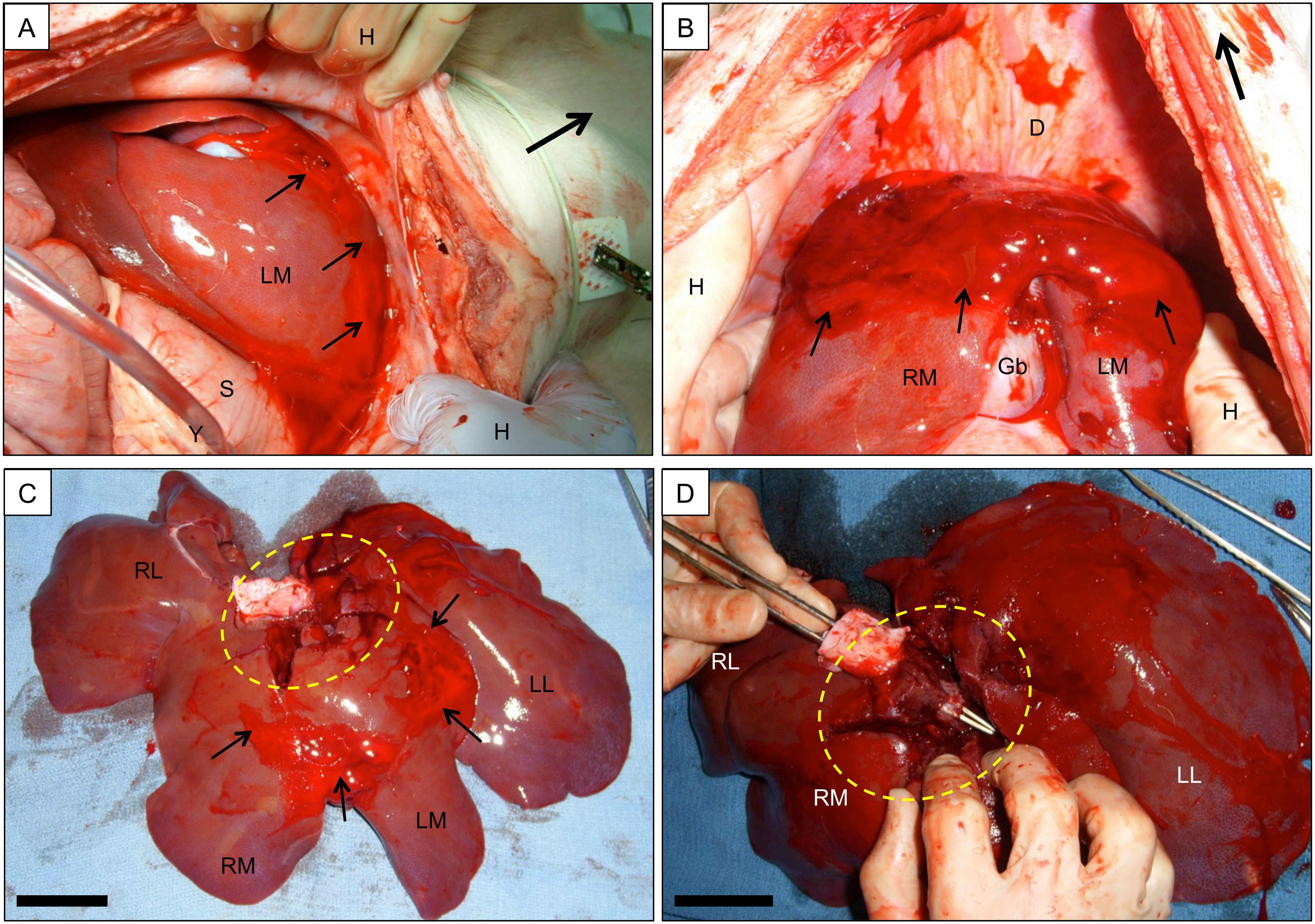

Figure S4, Yanala et al.

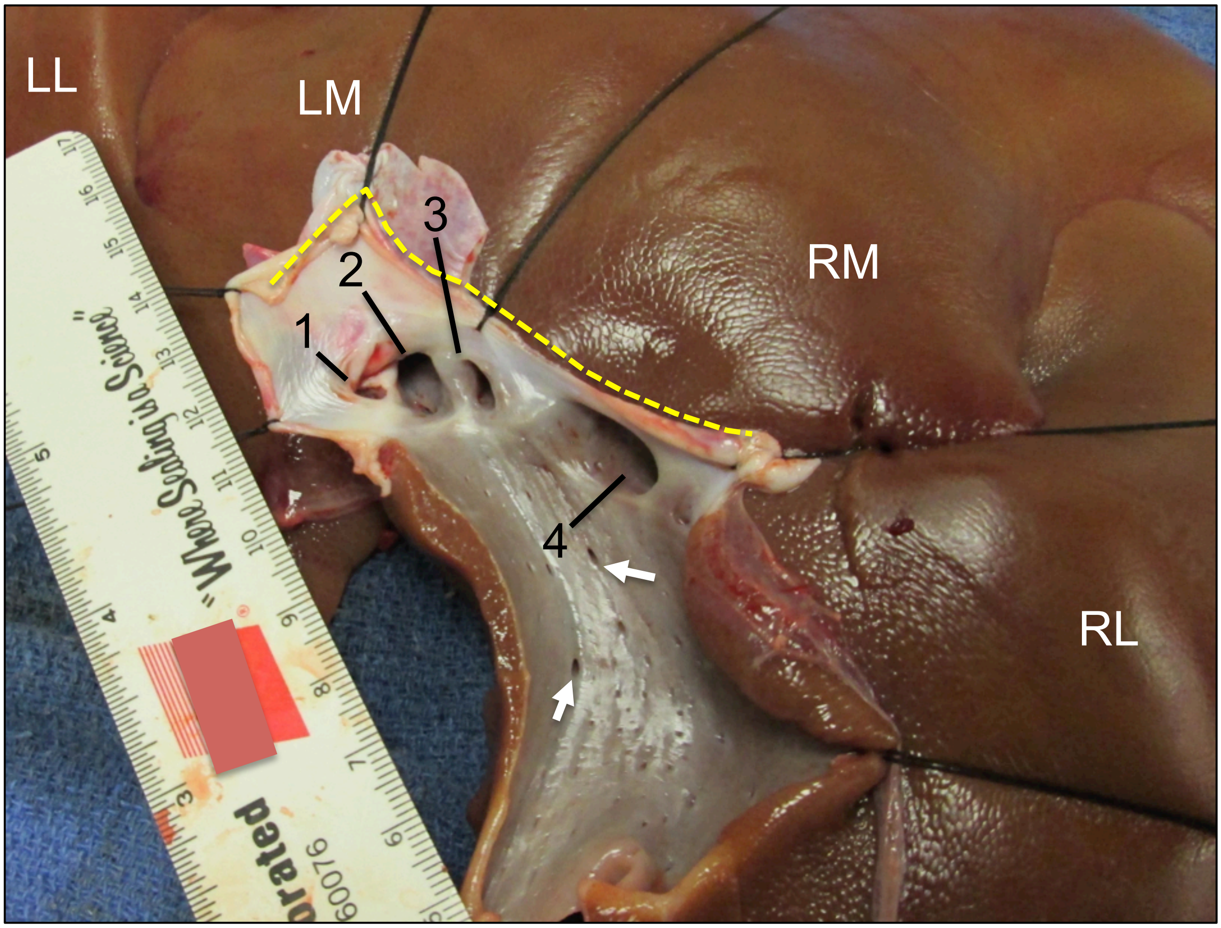

Figure S5, Yanala et al.

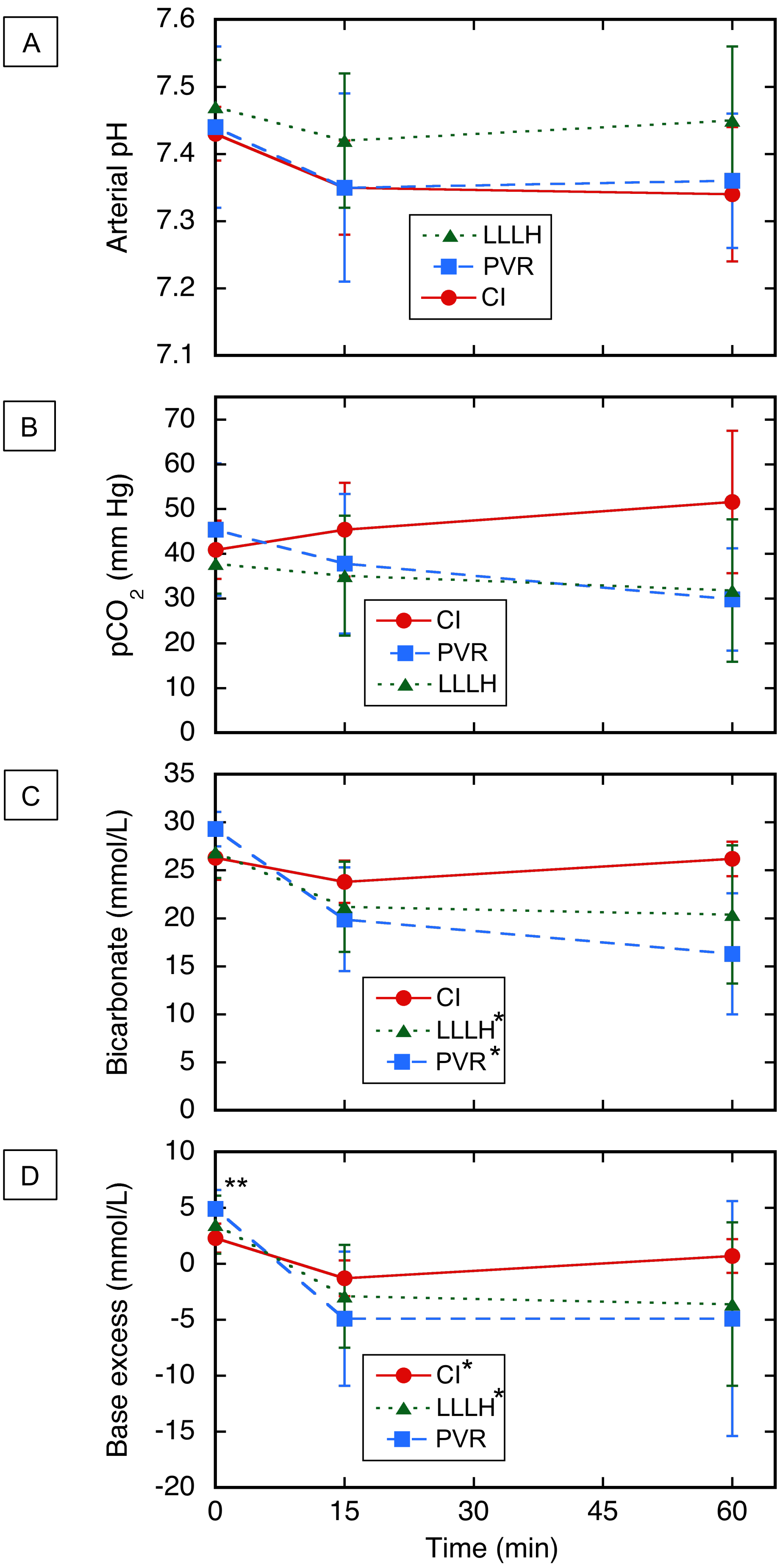

Figure S6, Yanala et al.

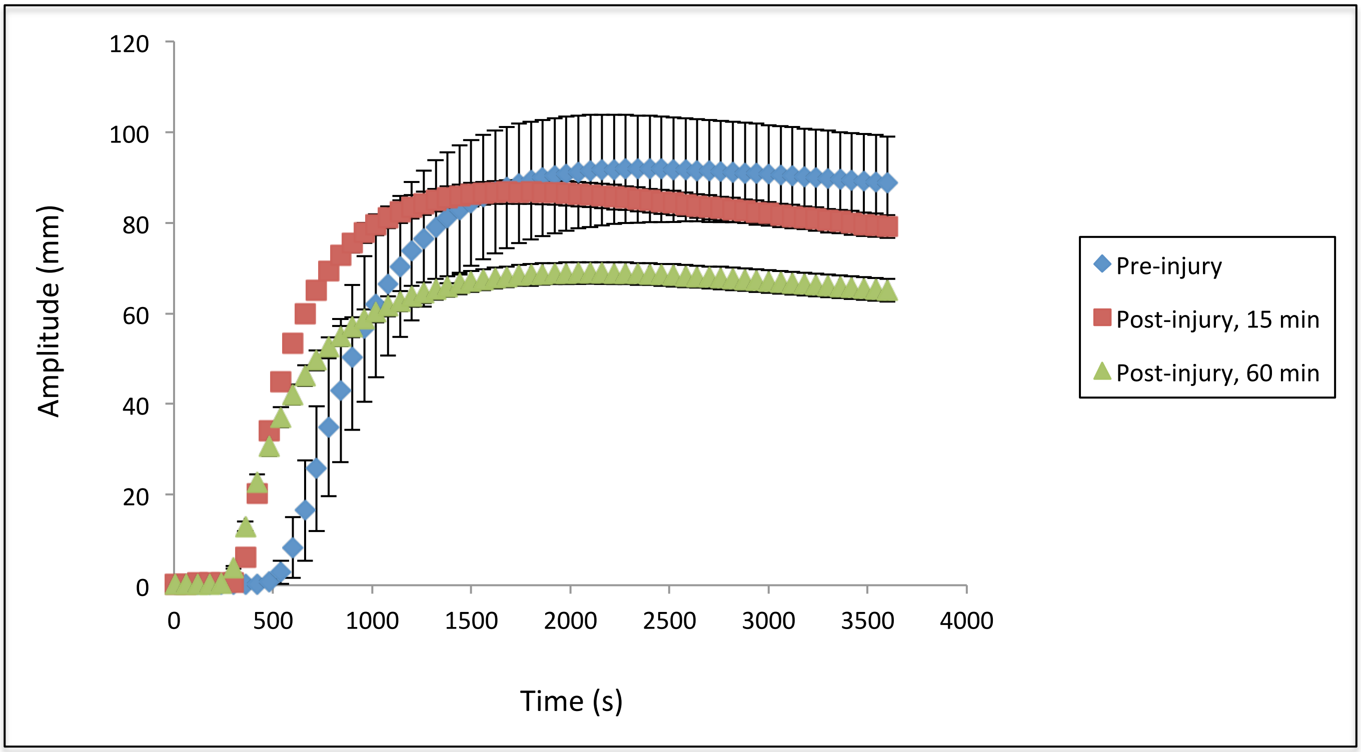

Figure S7, Yanala et al.

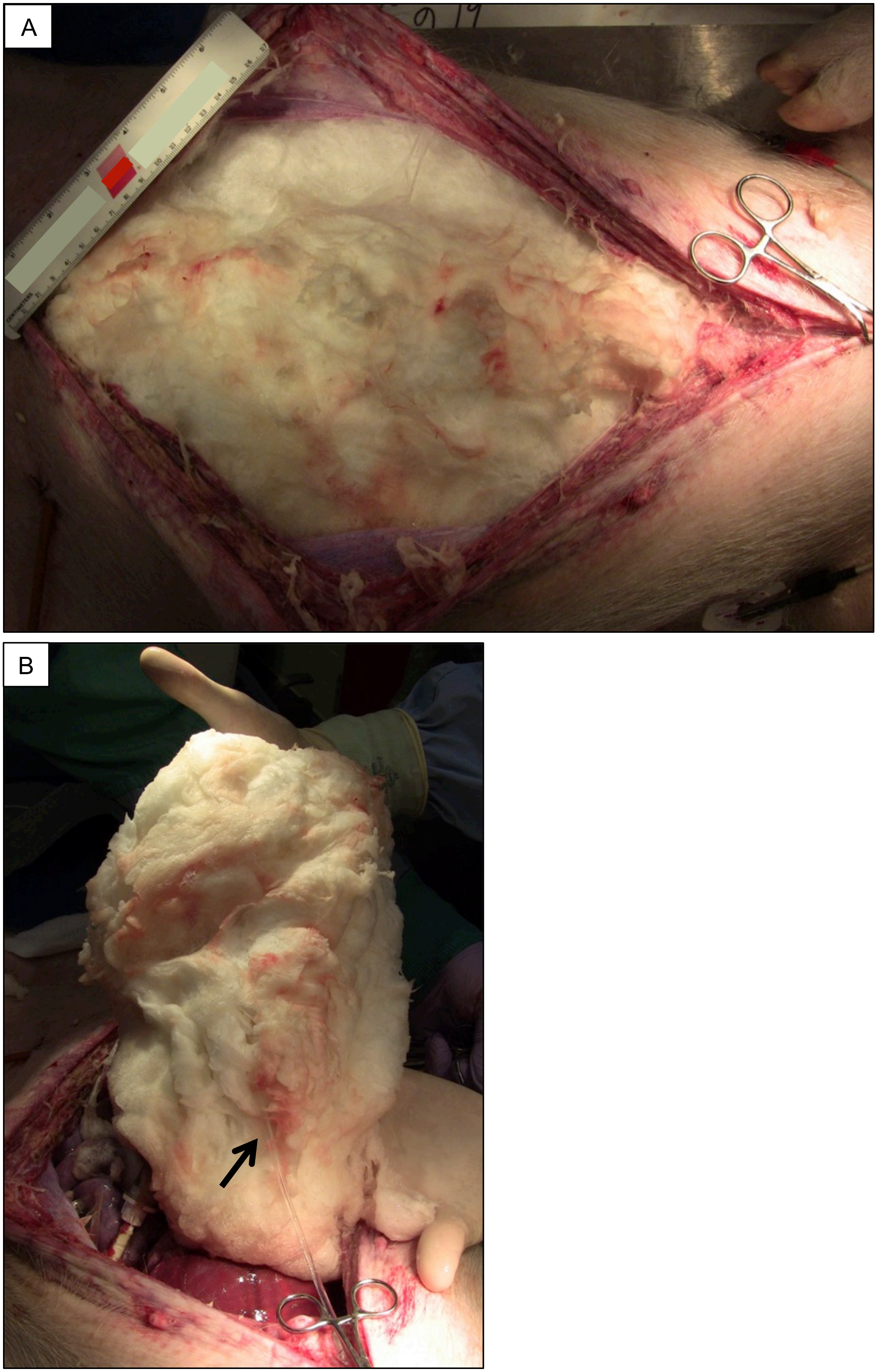

Figure S8, Yanala et al.
